# TMPRSS2 and MSPL Facilitate Trypsin-Independent Porcine Epidemic Diarrhea Virus Replication in Vero Cells

**DOI:** 10.3390/v9050114

**Published:** 2017-05-18

**Authors:** Wen Shi, Wenlu Fan, Jing Bai, Yandong Tang, Li Wang, Yanping Jiang, Lijie Tang, Min Liu, Wen Cui, Yigang Xu, Yijing Li

**Affiliations:** 1 College of Veterinary Medicine, Northeast Agricultural University, Harbin 150030, China; wenshi_china@163.com (W.S.); fanwenlu1230@163.com (W.F.); bj0815@126.com (J.B.); wanglicau@163.com (L.W.); jiangyanping2017@126.com (Y.J.); tanglijie@neau.edu.cn (L.T.); cuiwen_200@163.com (W.C.); 2 Harbin Veterinary Research Institute, Chinese Academy of Agricultural Sciences, Harbin 150001, China; tangyandong2008@163.com; 3 College of Animal Science and Technology, Northeast Agricultural University, Harbin 150030, China; liumin-707@163.com

**Keywords:** porcine epidemic diarrhea virus, type II transmembrane serine protease, TMPRSS2, MSPL, virus replication

## Abstract

Type II transmembrane serine proteases (TTSPs) facilitate the spread and replication of viruses such as influenza and human coronaviruses, although it remains unclear whether TTSPs play a role in the progression of animal coronavirus infections, such as that by porcine epidemic diarrhea virus (PEDV). In this study, TTSPs including TMPRSS2, HAT, DESC1, and MSPL were tested for their ability to facilitate PEDV replication in Vero cells. Our results showed that TMPRSS2 and MSPL played significant roles in the stages of cell–cell fusion and virus–cell fusion, whereas HAT and DESC1 exhibited weaker effects. This activation may be involved in the interaction between TTSPs and the PEDV S protein, as the S protein extensively co-localized with TMPRSS2 and MSPL and could be cleaved by co-expression with TMPRSS2 or MSPL. Moreover, the use of Vero cells expressing TMPRSS2 and MSPL facilitated PEDV replication in the absence of exogenous trypsin. In sum, we identified two host proteases, TMPRSS2 and MSPL, which may provide insights and a novel method for enhancing viral titers, expanding virus production, and improving the adaptability of PEDV isolates in vitro.

## 1. Introduction

Porcine epidemic diarrhea (PED) is caused by porcine epidemic diarrhea virus (PEDV) and is an acute and highly contagious enteric viral disease in nursing pigs. It is characterized by vomiting and lethal, watery diarrhea, and is becoming a global problem [1,2,3,4,5,6,7]. PED was first reported in feeder pigs and fattening swine in the United Kingdom in 1971 [8]. Since then, the disease has emerged in many pig-producing countries in Europe and Asia, resulting in tremendous economic losses to the pig industry. PEDV mainly infects the villous epithelial cells of the small intestine, which are rich in proteases, and causes atrophy of the villi, resulting in dehydration and diarrhea. Currently, although PEDV can be propagated in Vero cells treated with trypsin, which mediates the activation of virions for membrane fusion by cleaving the spike (S) glycoprotein [9,10], propagation of PEDV in vitro in a more productive manner remains a continued challenge. Sometimes, PEDV that has been isolated from clinical samples gradually loses its infectivity during further passages in cell cultures [1]. Therefore, the development of novel strategies to control PEDV is urgently required.

PEDV is a group I coronavirus (CoV) consisting of an enveloped virus with a single-stranded, positive-sense RNA genome of approximately 30 kb [11]. The S glycoproteins of CoVs are class I fusion proteins that are generated in a locked conformation to prevent premature triggering of the fusion mechanism and are subsequently prepared for action by proteolytic processing in a step called priming [12,13]. The S protein can be cleaved by endogenous proteases, which is thought to be necessary for inducing cell–cell fusion and virus-cell fusion [12,14,15,16,17]. Some endogenous proteases present in the pig small intestine potentially facilitate the entry of PEDV virions into intestinal epithelial cells [18]. However, in vitro, PEDV-infected cells produce syncytia only after treatment with an exogenous protease such as trypsin. This exogenous protease cleavage event leads to cell–cell and virus–cell fusion [14,16,19,20,21]. Therefore, the proteases responsible for PEDV activation may be potential therapeutic targets.

Recently, a type of trypsin-like serine protease termed type II transmembrane serine proteases (TTSPs) was reported to cleave and activate influenza virus and coronavirus surface proteins, allowing multicycle replication in the absence of trypsin. As previously described, transmembrane protease serine 2 (TMPRSS2) and human airway trypsin-like protease (HAT) can facilitate the spread of human influenza viruses [22,23,24,25]. TMPRSS2 and TMPRSS4 play important roles in influenza virus replication, supporting the spread of influenza virus in the absence of trypsin [26]. Subsequent studies confirmed that TMPRSS2 also can activate the spike protein of human coronaviruses, such as severe acute respiratory syndrome coronavirus (SARS-CoV) [27,28,29] and Middle East respiratory syndrome coronavirus (MERS-CoV) [30,31]. Zmora et al. evaluated seven TTSPs previously reported to activate the surface proteins of influenza A viruses (FLUAVs), MERS-CoV, and SARS-CoV and found that mosaic serine protease large-form (MSPL) and differentially expressed squamous cell carcinoma gene 1 (DESC1) contributed to viral spread in the host [32]. Moreover, the role of TMPRSS2 in the release of PEDV from infected cells was clarified [33]. However, the effects of transmembrane serine proteases on host infection by animal coronaviruses, especially PEDV, have not been thoroughly studied thus far.

In this study, to explore the mechanism of PEDV infection, optimize culture methods, and improve the proliferation of PEDV in vitro, the TTSPs TMPRSS2, HAT, DESC1, and MSPL were assessed to determine their effects on PEDV replication in Vero cells. The results may provide a novel approach to propagating PEDV in vitro as well as potential therapeutic targets for controlling PEDV infection.

## 2. Materials and Methods

### 2.1. Plasmids and Primers

pDONR223 plasmids containing the TMPRSS2 gene (BC051839), HAT gene (BC125195), DESC1 gene (BC113412), and MSPL gene (BC114928) were kindly provided by Biogot Technology, Public Protein/Plasmid Library, Nanjing, China. Recombinant pCMV-Myc plasmids expressing human TMPRSS2, HAT, DESC1, or MSPL were constructed following gene amplification and digestion by EcoRI and BglII. All polymerase chain reaction (PCR) primers used in this study are listed in Table 1.

### 2.2. Cell and Virus Culture

The swine intestinal epithelial cell (IEC) line [34,35,36,37] and Vero cells (ATCC, Manassas, VA, USA) were cultured in Dulbecco’s modified Eagle medium (DMEM; Gibco, Grand Island, NY, USA) containing 10% fetal calf serum (FCS; Gibco). Cell-adapted PEDV strain LJB/03 from our laboratory [38,39,40] was propagated in Vero cells and IECs. Briefly, the confluent cell monolayer was washed once with sterile phosphate-buffered saline (PBS) and incubated at 37 °C for 1 h with PEDV LJB/03 supplemented with 21 μg/mL trypsin; then, the inoculum was removed, the cells were washed twice with PBS, and the maintenance medium (DMEM) was supplemented with 5 μg/mL trypsin. Cell cultures were harvested until the cytopathic effect (CPE) exceeded 80%. After freeze-thaw treatment, the supernatants were collected and stored at −80 °C until required.

### 2.3. Expression of TTSPs in Transfected Vero Cells

Vero cells were transfected with pCMV-Myc expressing TMPRSS2, HAT, DESC1, or MSPL, using Lipofectamine LTX & Plus Reagent (Invitrogen, Life Technologies, Carlsbad, CA, USA). Then, 3 μg/well of recombinant plasmid DNA was diluted into 500 μL of Opi-MEM I reduced-serum medium (Gibco) without serum and mixed with an equal volume of PLUS reagent gently. The mixture was incubated at room temperature (RT; 20–25 °C) for 5 min. Lipofectamine LTX was added, and the complexes were allowed to form by incubation for 30 min. The DNA-Lipofectamine LTX complexes were then added to each well containing cells and medium. In parallel, Vero cells transfected with the same concentration of empty plasmid were used as a control. Post-transfection cells were cultured in 6-well plates at a density of 1.5 × 10^5^/well and cultivated for 48 h. For analysis of TTSP expression by immunofluorescence, cells were washed three times with PBS and fixed with 4% paraformaldehyde at RT for 15 min; then, the cells were permeabilized with 0.2% Triton X-100 in PBS at RT for 10 min and blocked in PBS with 0.3% bovine serum albumin at 37 °C for 30 min. Subsequently, the cells were treated with mouse anti-Myc antibody (Sigma, St. Louis, MO, USA) and rhodamine Red-X-coupled anti-mouse (ZSGB-BIO, Beijing, China) as primary and secondary antibodies, respectively, followed by counterstaining with 4′,6-diamidino-2-phenylindole (DAPI, Beyotime, Shanghai, China). Then, a fluorescence microscope (Leica, Wetzlar, Germany) was used to visualize staining. For analysis of TTSP expression by western blot, cells were washed with PBS, and detached with 200 μL of cell lysis buffer (Beyotime) containing 1 mM phenylmethanesulfonyl fluoride (PMSF; Beyotime). Cells were subjected to sonication, mixed with 5× sodium dodecyl sulfate (SDS) loading buffer, and denatured in boiling water for 10 min. Following SDS-polyacrylamide gel electrophoresis (SDS-PAGE), proteins were transferred to a polyvinylidene fluoride membrane (Merck Millipore, Darmstadt, Germany), and immunoblots were developed using mouse anti-Myc antibody (Sigma) as the primary antibody and horseradish peroxidase (HRP)-conjugated goat anti-mouse antibody (Thermo, Waltham, MA, USA) as the secondary antibody. As a loading control, mouse anti-actin antibody (Sigma) was used. For analysis of TTSPs by flow cytometry, Vero cells transfected with TTSP-encoding plasmid were detached, washed with PBS, incubated with ice-cold ethanol for 10 min, and then stained with mouse anti-Myc primary antibodies (Sigma) followed by DyLight 647-coupled anti-mouse secondary antibodies (Dianova, BioLeaf Biotech, Shanghai, China). After three washings with PBS, cells were fixed with 2% paraformaldehyde and staining was analyzed with an Aria II flow cytometer (BD Biosciences, San Jose, CA, USA).

### 2.4. Quantitative Real-Time PCR Analysis

The viral RNA of PEDV propagated in Vero cells was extracted using E.Z.N.A. Total RNA Kit I (Omega Bio-Tek, Doraville, GA, USA) following the manufacturer’s protocol. Complementary DNA (cDNA) was produced via reverse transcription, using oligo(dT)_15_ (Takara, Tokyo, Japan) and the Superscript Reverse Transcriptase Reagent Kit (Takara) according to the manufacturer’s instructions. Then, an ABI 7500 real-time PCR system (Applied Biosystems, Carlsbad, CA, USA) was used to determine viral mRNA transcript levels with SYBR Premix EX Taq II (Takara) according to the manufacturer’s recommendations. The specific real-time PCR primers targeting the *N* gene of PEDV and the β-actin gene of Vero cells are described in Table 1. Real-time PCR was performed under the following conditions: 40 cycles of 30 s at 95 °C, 3 s at 95 °C, and 30 s at 60 °C. The average cycle threshold (Ct) for each individual assay was calculated from triplicate measurements using the instrument’s software in “auto Ct” mode (ABI 7500 system software, version 2.3). Relative Ct values of three independent tests were calculated by the 2^−ΔΔCt^ method. Levels of *N* transcripts were normalized to those of β-actin transcripts in the same sample, and the 2^−ΔΔCt^ value of viral RNA in each sample was analyzed in parallel. There were no specific signals detected in any negative controls.

### 2.5. Determination of Viral Titer of PEDV Propagated in Vero Cells Expressing TTSPs

Prior to investigating the infectivity of PEDV LJB/03 propagated in Vero cells transiently expressing TTSPs, the viral titer was determined by plaque assay. In brief, after digestion, suspended Vero cells were transfected with 3 μg/well of pCMV-Myc plasmids expressing TMPRSS2, HAT, DESC1, or MSPL, with the empty pCMV-Myc plasmid used as a control. Then, the Vero cells were seeded into 6-well plates at 1.5 × 10^5^/well, and after 24 h, the cells were infected at a multiplicity of infection (MOI) of 0.1 in an infection medium with 3 μg/mL trypsin or PBS. After 1 h of viral adsorption, the inoculum was removed, and the cells were washed twice with PBS and fixed with 3 mL of Minimum Essential Medium (MEM, Gibco) with 0.8% agarose. When CPEs appeared, cells were stained with MEM containing 0.01% Neutral Red Solution (Sigma), and syncytia were counted as plaque under a microscope. The viral titer is expressed as plaque-forming units (PFU)/mL.

### 2.6. Determination of Effects of TTSPs and TTSP Inhibitor on Viral Replication

To analyze the effects of TTSPs on viral replication, the replication kinetics of intracellular viral RNA were determined by quantitative real-time PCR. Vero cells were transfected with 1 μg/well of pCMV-Myc plasmids expressing a TTSP (TMPRSS2, HAT, DESC1, or MSPL) or empty pCMV-Myc plasmid (control) and seeded in 24-well plates. Then, the cells were infected with PEDV at a multiplicity of infection (MOI) of 0.01 and supplemented with 3 μg/mL trypsin or PBS. After viral adsorption, the cells were washed twice with PBS and cultured with DMEM. At different time points post-infection, the cells were collected and subjected to quantitative real-time PCR detection as described above. To examine the viral replication in Vero cells treated with a TTSP inhibitor, TTSP-transfected Vero cells were pretreated with 200 μM or 500 μM of the TTSP inhibitor AEBSF-HCl (Sigma) or PBS for 1 h, as previously published [41]. Then, the treated cells were infected with PEDV LJB/03 at an MOI of 0.01 for 1 h; at 12 h post-infection, levels of viral replication were determined by quantitative real-time PCR.

### 2.7. Analysis of PEDV and TTSP Co-Localization

To determine the cellular localization of the S protein of PEDV and the TTSPs, Vero cells were transfected with pCMV-Myc plasmids expressing TMPRSS2, HAT, DESC1, or MSPL, or with empty plasmid serving as a negative control. At 24 h post-transfection, the cells were washed with PBS and infected with PEDV LJB/03 at an MOI of 1. The pCMV-Myc-transfected cells were infected with PEDV in the absence or presence of 3 μg/mL trypsin. At 24 h post-infection, the cells were fixed with 4% paraformaldehyde, permeabilized with 0.2% Triton X-100, and blocked with 0.3% bovine serum albumin. Then, the cells were incubated with mouse anti-Myc antibody (Sigma) and rabbit anti-PEDV S protein polyclonal antibody (developed in our laboratory) at RT for 1 h. After washing with PBS three times, the cells were incubated with fluorescein isothiocyanate (FITC)-conjugated goat anti-rabbit IgG (ZSGB-BIO) and Alexa Fluor 647-labeled goat anti-mouse IgG (H + L) (ZSGB-BIO) secondary antibodies at RT for 1 h. After washing, the cells were treated with DAPI (Beyotime). The coverslips were mounted on glass microscope slides in mounting buffer and examined using a laser scanning microscope (Leica TCS SP2, Wetzlar, Germany). Further image analysis, including calculation of the Pearson correlation coefficient (PCC), was performed with Image J with Just Another Colocalization Plugin [32,42].

### 2.8. Cleavage of PEDV S Protein by TTSPs

To determine the cleavability of S protein by TTSPs, PEDV strain LJB/03 S protein was cloned into plasmid pCMV-HA. Then, 293T cells were seeded into six-well plates at a density of 2 × 10^5^/well and cotransfected with 2 μg/well of plasmid encoding PEDV S with a N-terminal HA tag and 2 μg/well TTSPs-expressing plasmid or an empty plasmid by using Lipofectamine LTX & Plus Reagent (Invitrogen) according to the manufacturer’s protocol. At 48 h posttransfection, the cells were harvested and subjected to sonication and denatured. The lysates were separated by SDS-PAGE and blotted onto polyvinylidene fluoride membrane (Merck Millipore). The PEDV S protein with a N-terminal HA antigenic tag was detected by staining with mouse monoclonal antibody specific for the HA tag (Sigma), followed by incubation with an HRP-conjugated goat anti-mouse antibody (Thermo). As a loading control, expression of β-actin was detected with an anti-β-actin antibody (Sigma).

### 2.9. Analysis of TTSP Activation of PEDV for Cell–Cell Fusion

To determine the effects of TTSPs on PEDV for cell-cell fusion, the CheckMate Mammalian Two-Hybrid System (Promega, Madison, WI, USA) was used. In brief, Vero target cells were transfected with either empty pCMV-Myc plasmid or pCMV-Myc plasmids encoding TTSPs in combination with the pG5-luc plasmid, which carries the firefly luciferase reporter gene under the control of a promoter containing five GAL4-binding sites. In parallel, Vero effector cells were transfected with the plasmids pACT (containing the herpes simplex virus VP16 activation domain upstream of a multiple cloning region) and pBind (expressing Renilla reniformis luciferase under the control of the SV40 promoter). After 24 h, the effector cells were detached, diluted in fresh medium, and added to the target cells. After 24 h of co-cultivation, the cells were washed with PBS and infected with PEDV LJB/03 at an MOI of 1 and supplemented with 1 μg/mL or 0.1 μg/mL trypsin or PBS. Cell–cell fusion was quantified by determining luciferase activity in cell lysates with a commercially available kit (Promega) after 48 h of co-cultivation.

### 2.10. Quantitative Analysis of TTSP Expression in the Normal and PEDV-Infected Piglet Small Intestine/IECs

Total RNA samples obtained from small intestine tissues of three normal and three PEDV-infected piglets were used to quantify gene expression levels of TTSPs. cDNA was produced using Superscript Reverse Transcriptase Reagent Kit (Takara) according to the manufacturer’s instructions, and a quantitative real-time PCR assay was performed in triplicate with SYBR^®^ Premix EX Taq II (Takara) using the *GAPDH* gene as a control. The primers used are shown in Table 1. Average Ct values calculated for *TMPRSS2*, *HAT*, *DESC1,* and *MSPL* were normalized by subtraction from the Ct values obtained for *GAPDH* as an internal control. Template-free cDNA reaction mixtures were analyzed in parallel, and no specific signal was detected in any of these experiments. The piglets were handled and maintained under strict ethical considerations according to international recommendations for animal welfare. In addition, the TTSP expression levels in the normal IECs and PEDV-infected IECs were also subjected to quantitative real-time PCR detection as described above.

### 2.11. Adaptation of PEDV Isolated from Clinical Samples to Vero Cells Transiently Expressing TTSPs

To analyze the adaptation of PEDV strains isolated from clinical samples to Vero cells transfected with pCMV-Myc plasmids expressing TTSPs, two PEDV-positive small intestine tissue samples (A and B) collected from outbreaks of severe acute diarrhea in suckling piglets in 2013 and 2014 in China were tested. Twenty-four hours after transfection with plasmids expressing TTSPs or empty plasmid (control), Vero cells were infected with processed viral samples supplemented with PBS or 3 μg/mL trypsin for 72 h. Following three serial passages, total RNA was extracted to assess the relative RNA levels of PEDV by qPCR.

### 2.12. Statistical Analysis

All experiments were repeated 3–5 times. Data were statistically analyzed by one-way ANOVA, using GraphPad Prism v5.0 software. *p* < 0.05 was considered statistically significant.

## 3. Results

### 3.1. Expression of TTSPs in Transfected Vero Cells

**Figure 1:**
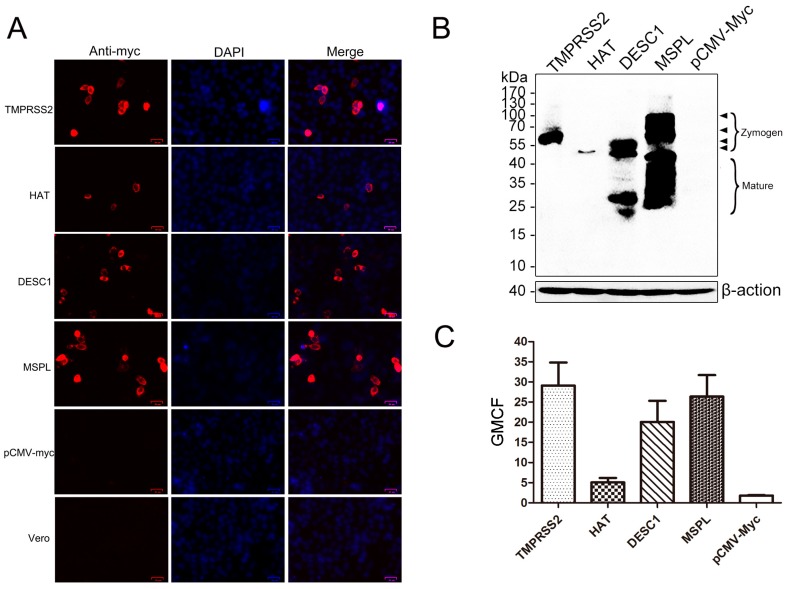
Expression of type II transmembrane serine proteases (TTSPs) in transfected Vero cells. (**A**) Post-transfection, the expression of TMPRSS2, HAT, DESC1, and MSPL in transfected Vero cells was detected via indirect immunofluorescence. Bar = 25 μm. Magnification, ×200; (**B**) TTSP expression in transfected Vero cells as determined by western blot. Zymogens and the mature form are indicated; (**C**) TTSPs expression was detected by FACS. The geometric mean channel fluorescence (GMCF) measured in a representative experiment performed with triplicate samples is shown. Error bars indicate standard deviations of three independent experiments.

The genes encoding TMPRSS2, HAT, DESC1, and MSPL were cloned into pCMV-Myc plasmids and transfected to Vero cells individually. Expression of the four TTSPs in transfected Vero cells was detected via indirect immunofluorescence, western blot assay and fluorescence-activated cell sorting (FACS). As shown in Figure 1A,B, the four TTSPs were successfully expressed in Vero cells transiently transfected with pCMV-Myc plasmids expressing TMPRSS2, HAT, DESC1, or MSPL. TTSPs were expressed at the cellular plasma membrane, and the number of HAT-positive cells was lower than that for the other three TTSP-positive cells (Figure 1A). The expression of most proteases was readily detectable with the proper predicted size for each, as previously published [32], but the proteases were not expressed in Vero cells transfected with empty pCMV-Myc plasmid (Figure 1B). TTSPs are synthesized as inactive single-chain zymogens and undergo self-cleavage into active forms during or after transport to cell surfaces [43,44]. DESC1 and MSPL were found to be activated and to form bands presenting the cleaved catalytic domain of mature forms. Moreover, we analyzed TTSP expression levels by fluorescence-activated cell sorting (FACS) of stained cells. The most prominent signal was measured in TMPRSS2-expressing cells, followed by MSPL- and DESC1-expressing cells. The fluorescence signal obtained from HAT-expressing cells was the weakest (Figure 1C).

### 3.2. Effects of TTSPs and TTSP Inhibitor on Viral Replication

**Figure 2:**
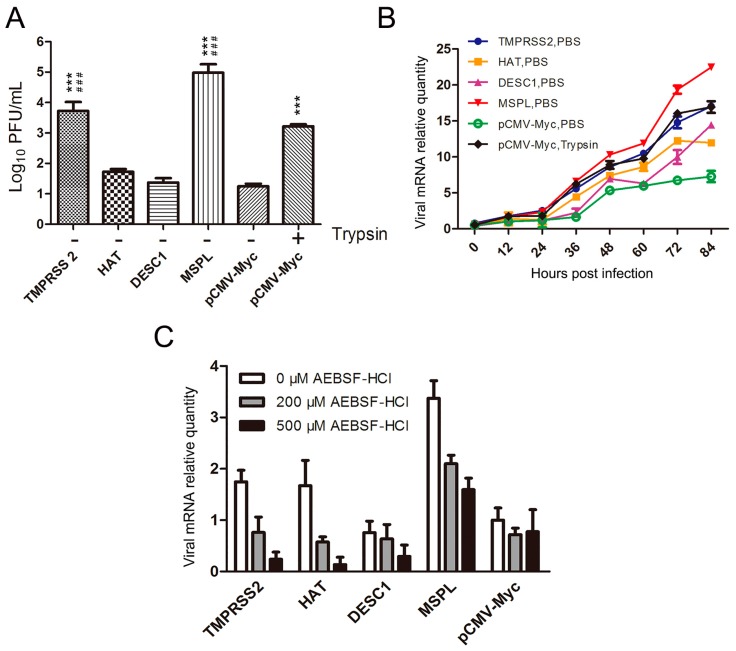
Effects of TTSPs and TTSP inhibitor on viral replication. (**A**) Porcine epidemic diarrhea virus (PEDV) titers following the expression of TTSPs in Vero cells. Viral titers were determined by plaque assay. *** *p* < 0.001 vs. empty pCMV-Myc plasmid; ^##^^#^ *p* < 0.001 vs. empty pCMV-Myc plasmid with 3 μg/mL trypsin; (**B**) Replication kinetics of intracellular viral RNA in Vero cells expressing TTSPs. Relative quantity of the empty pCMV-Myc plasmid with PBS at 0 h = 1; (**C**) Viral replication after TTSP inhibitor treatment. Error bars indicate the standard error of three independent experiments. The relative quantity of the empty pCMV-Myc plasmid with 0 μM AEBSF-HCl treatment = 1.

Prior to this investigation, the presence of endogenously expressed TTSPs in Vero cells was analyzed by RT-PCR assay with primers targeting monkey-borne TMPRSS2 (XM_007968781), HAT (XM_007998573), DESC1 (XM_007998564), and MSPL (XM_008021030) genes (Table 1). No *TMPRSS2*, *HAT*, *DESC1*, or *MSPL* mRNA was detected in the Vero cells in this study. Next, the effects of these proteases on PEDV replication in Vero cells exogenously expressing TTSPs were examined. Following transfection, Vero cells were infected with PEDV in the presence or absence of trypsin. As shown in Figure 2A, in the absence of trypsin, the viral titers of PEDV propagated in Vero cells transfected with pCMV-Myc expressing TMPRSS2 and MSPL were clearly higher than those of PEDV propagated in Vero cells expressing HAT and DESC1. Among the TTSPs, the viral titers in Vero cells expressing TMPRSS2 and MSPL were almost 10^2.5^ to 10^4.5^ times higher than those in the empty-plasmid group and even 3- to 30-fold higher than those in Vero cells cultured with trypsin (3 μg/mL). Moreover, the viral RNA levels in each group were determined by qPCR at different time points post-infection. As shown in Figure 2B, at 72 h post-infection, viral RNA levels in Vero cells expressing MSPL were significantly higher than those in the other groups, and the viral mRNA relative quantity in trypsin-treated cells was slightly higher than that in TMPRSS2-transfected cells. However, the efficacy of TMPRSS2 in activating PEDV replication was almost the same as that of 3 μg/mL trypsin and was higher than that of HAT or DESC1 at 84 h post-infection. These findings indicate that MSPL and TMPRSS2 play important roles in PEDV infection.

Furthermore, the TTSP inhibitor AEBSF-HCl was used to evaluate the effects of TTSPs on trypsin-independent PEDV entry. The cytotoxicity of AEBSF-HCl at the recommended concentrations was first tested to exclude cytotoxic effects. Then, TTSP-transfected Vero cells were treated with AEBSF-HCl and infected with PEDV. The pCMV-Myc-transfected cells were infected with PEDV in the absence of trypsin. At 12 h post-infection, viral RNA levels were determined by qPCR. As shown in Figure 2C, AEBSF-HCl induced strong inhibitory activity, resulting in dose-dependent decreases in the viral RNA levels. The viral RNA levels of PEDV in MSPL-transfected Vero cells treated with 500 μM AEBSF-HCl were significantly higher than those of cells transfected with other TTSPs. These results also indicate that TTSPs such as TMPRSS2 and MSPL play an important role in PEDV entry, suggesting that TMPRSS2 and MSPL promote PEDV replication better than trypsin.

### 3.3. TTSP Activation of PEDV for Cell–Cell Fusion

**Figure 3:**
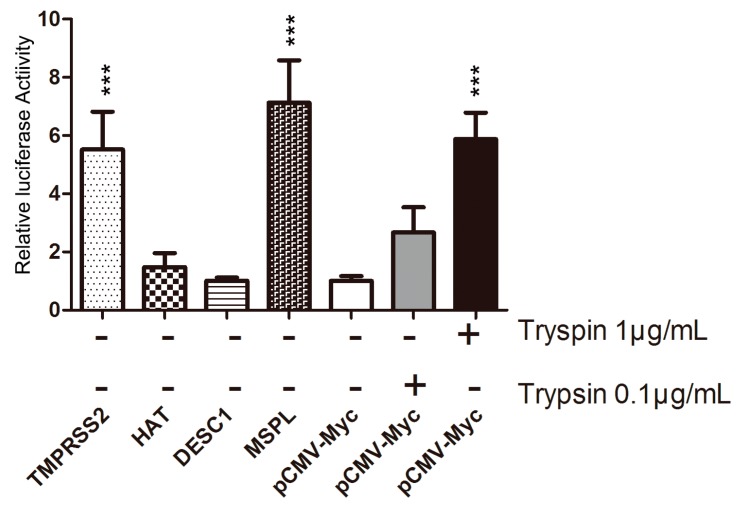
TMPRSS2 and MSPL activation of PEDV for cell–cell fusion. The results of a representative experiment performed with triplicate samples are shown; *** *p* < 0.001 vs. pCMV-Myc without trypsin. Relative quantity of pCMV-Myc without trypsin = 1. Error bars indicate standard error of the mean.

We evaluated the impact of TTSP expression on PEDV-infected Vero cells, using a cell–cell fusion assay (Figure 3). Among the four TTSPs, the expression of MSPL and TMPRSS2 in Vero target cells significantly promoted fusion with Vero effector cells following PEDV infection; in particular, MSPL facilitated cell fusion better than 1 μg/mL trypsin treatment. In contrast, transfection with HAT and DESC1 did not promote cell–cell fusion that was observed with the empty plasmid control. These results indicate that MSPL and TMPRSS2 facilitate PEDV replication.

### 3.4. Co-Localization of TTSPs and PEDV

**Figure 4:**
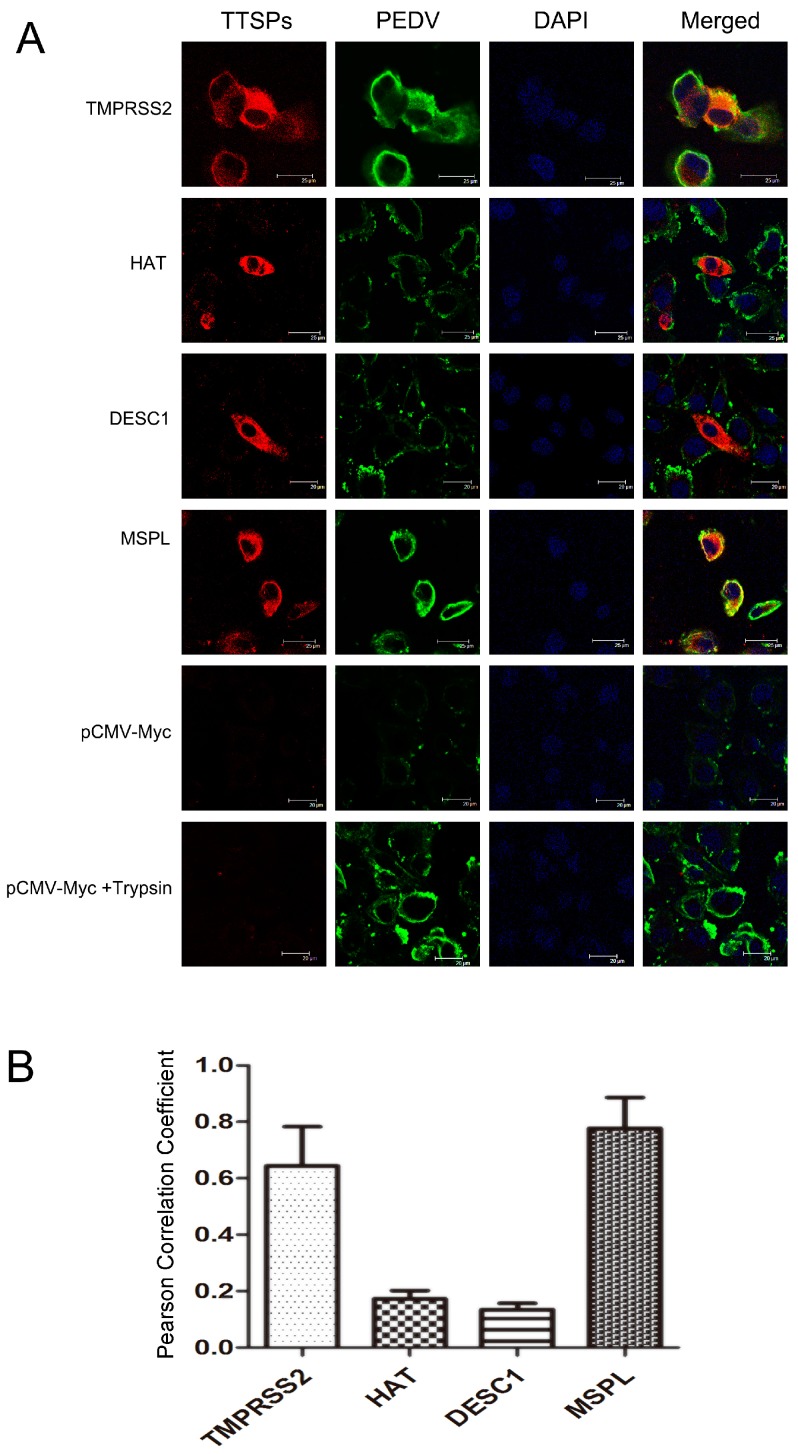
Analysis of TTSP and PEDV S protein co-localization. (**A**) Analysis of TTSP and PEDV S protein co-localization using a laser scanning microscope. Bar = 20–25 μm. Magnification, ×400; (**B**) The co-localization of TTSPs and S protein was determined by calculation of Pearson correlation coefficient (PCC). The average PCC measured for three to five cells from separate experiments is shown; error bars indicate the standard errors of the means.

In this study, the co-localization of the four TTSPs with the PEDV S protein was investigated in infected Vero cells to determine the mechanism of PEDV activation by TTSPs. As shown in Figure 4A, immunofluorescence staining of TTSP-transfected Vero cells infected with PEDV revealed that the PEDV S protein was extensively co-localized with MSPL and TMPRSS2 but not with HAT or DESC1. This assessment was confirmed upon determination of the PCC for TTSPs and S protein signals. The S signals correlated well with those of TMPRSS2 and MSPL, indicating extensive co-localization, whereas little correlation was measured for the S protein and HAT or DESC1 signals (Figure 4B). Thus, the cellular localizations of S protein and TMPRSS2 or MSPL overlap extensively, indicating that MSPL and TMPRSS2 may interact with S protein, activating PEDV replication in Vero cells.

### 3.5. Effects of TTSPs on PEDV S Protein Cleavage

**Figure 5:**
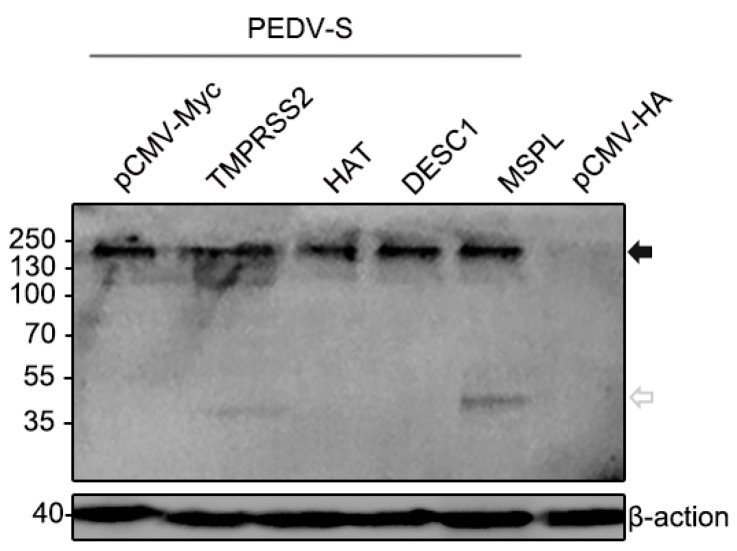
TMPRSS2 and MSPL cleave the PEDV S protein. Black-filled arrowheads, uncleaved S protein; white-filled arrowheads, N-terminal cleavage fragments.

We further assessed if the TTSPs studied were able to cleave the S protein of PEDV. As shown in Figure 5, the full-length PEDV S proteins migrating at 200 kDa were detected using anti-HA antibody reacting with the N-terminal of the PEDV S protein. Cleavage of PEDV S was detected upon the coexpression of TMPRSS2 and MSPL. The size of cleavage fragments were the same, approximately 35 kDa. In contrast, coexpressing of HAT or DESC1 did not facilitate PEDV S cleavage. Shirato et al. found that PEDV S protein could be cleaved by co-expression with TMPRSS2, the cleavage C-terminal fraction of S protein detected was 160 kDa [33]. Therefore, our study further confirmed the roles of TMPRSS2 and MSPL in the PEDV S protein activation. The effects of TMPRSS2 and MSPL on PEDV S protein cleavage may be responsible for facilitating the replication of PEDV.

### 3.6. Determination of TTSP Expression in the Normal and PEDV-Infected Piglet Small Intestine/IECs

**Figure 6:**
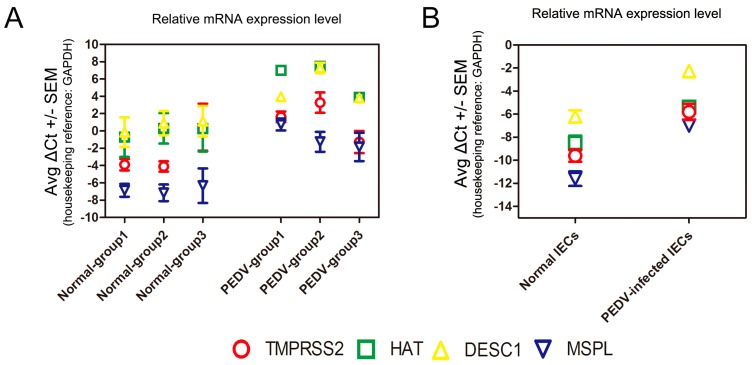
Expression of TTSPs in the normal and PEDV-infected porcine small intestine/intestinal epithelial cells (IECs). (**A**) Expression of TTSPs in the normal and PEDV-infected porcine small intestine; (**B**) Expression of TTSPs in the normal and PEDV-infected IECs.

We performed real-time RT-PCR analysis of the mRNA levels of TMPRSS2, HAT, DESC1, and MSPL in the small intestine tissues of normal and PEDV-infected piglets. The expression of DESC1 in the normal piglets was the highest, followed by HAT, TMPRSS2, and MSPL; moreover, the mRNA levels of all TTSPs increased in the small intestine of PEDV-infected piglets (Figure 6A). We also detected the TTSP level in IECs after PEDV infection. The endogenous TTSP level is up-regulated in IECs after PEDV infection, which was similar to that in piglet small intestine tissues infected with PEDV (Figure 6B). These results suggest that TMPRSS2, MSPL, HAT and to a higher degree, DESC1 are expressed in piglet small intestine, and that the endogenous TTSP level is up-regulated after PEDV infection. However, whether the endogenous presence of TTSPs in the small intestines of piglets contributes to viral spread in infected piglets remains to be determined.

### 3.7. TTSPs Facilitate Propagation of PEDV Isolates in Vero Cells

**Figure 7:**
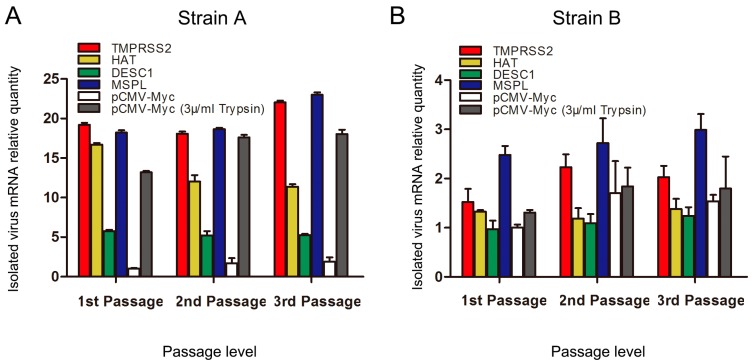
Culture of PEDV isolated from pig intestine in Vero cells transiently expressing TTSPs in three serial passages. (**A**) Isolation of PEDV strain A; (**B**) Isolation of PEDV strain B. The relative quantity of pCMV-Myc without trypsin at the 1st passage = 1. Error bars indicate standard error of the mean. Results shown are those of a representative experiment performed with triplicate samples.

Two PEDV-positive piglet small intestine samples were used to test the abilities of TMPRSS2, HAT, DESC1, and MSPL to facilitate PEDV. TTSP-transfected Vero cells were infected with processed viral samples, and the relative RNA levels of PEDV propagated in Vero cells expressing TMPRSS2, MSPL, HAT, or DESC1 were determined for three serial passages. As shown in Figure 7A, TMPRSS2 and MSPL facilitated strain A replication to an almost 20-fold higher extent than the TTSP control (pCMV-Myc group) after three serial passages. Additionally, HAT could also promote culture of strain A in Vero cells. We speculate that there may be an active site of HAT on the S protein of strain A. The effect of trypsin on the isolation of strain B was not significant. However, the effect of strain B cultured in Vero cells expressing TMPRSS2 and MSPL was better than trypsin treatment; the viral mRNA level in Vero cells expressing MSPL was two times higher than that of the trypsin group after three serial passages (Figure 7B). TMPRSS2 and MSPL facilitated the propagation of the two PEDV isolates (strains A and B) in Vero cells efficiently and steadily, suggesting a promising approach for PEDV propagation of clinical samples in the absence of trypsin treatment.

## 4. Discussion

During the 1970s and 1990s, PEDV caused widespread epidemics in multiple swine-producing countries in Europe [5,45,46,47]. Since then, severe outbreaks have emerged in a number of Asian countries, including Japan [48], China [49], South Korea [50], and Thailand [51]. Recently, PEDV has been spreading rapidly among swine farms in the United States, resulting in high piglet mortality in more than 32 states [52,53], and similar outbreaks have also been reported in Canada and Mexico [1,3,4,6]. Currently, severe PED is one of the most important diseases affecting pig farming in China [54]. However, the effectiveness of the CV777-based vaccine has been questioned because PED outbreaks have also occurred in vaccinated herds [55]. Therefore, there is an urgent need to improve the protective efficacy of vaccines and to develop new vaccines. However, PEDV isolation in vitro remains challenging, as the isolated virus may gradually lose infectivity upon continued passaging in cell cultures supplemented with trypsin [1]. Recently, we attempted to isolate PEDV from clinical samples, using porcine intestinal epithelial cells (IECs) and found that PEDV isolates were better adapted to growth in IECs than in Vero cells [34], indicating that some trypsin-like proteases present in the IECs facilitated the propagation of PEDV. Moreover, previous research has suggested that several TTSPs located in the mucosal epithelium play critical roles in viral infectivity through the activation of viral surface proteins [23,27,31,32]. At the beginning of this study, we designed primers according to the predicted sequences of swine TTSPs from national center for biotechnology information (NCBI), and attempted to amplify the full-length TTSP genes from the trachea, bronchus, lung, and small intestine tissues of piglet and IECs. However, we failed to obtain the porcine TTSP genes. We speculate that differences may exist between the actual sequences and predicted sequences. Although TTSPs are the host proteases of respiratory and digestive tract mucosa, the TTSP expression levels may be low or limited in some conditions or over a period of time, which results in difficulty in obtaining actual porcine TTSP genes. Therefore, we studied human TTSPs (TMPRSS2, HAT, DESC1, and MSPL) to explore their effects on PEDV replication.

The TTSP family is composed of more than 20 members and divided into four subfamilies: the HAT/DESC subfamily, hepsin/TMPRSS/enteropeptidase subfamily (including TMPRSS2 and MSPL), matriptase subfamily, and corin subfamily. TMPRSS2 and MSPL are predominantly expressed in the fetal liver and kidney [56] and on the brush-border of the duodenum [57]. HAT is predominantly expressed in the trachea [58,59], whereas DESC1 is restricted to the epithelia of the skin and oral cavity [60,61]. In this study, we confirmed the presence of TMPRSS2, HAT, DESC1, and MSPL in the small intestines of normal piglets and IECs, and we also found that the mRNA levels of these TTSPs increased in PEDV-infected small intestine tissues and IECs. Whether or not the endogenous presence of TTSPs in the small intestines of piglets contributes to viral spread in infected piglets remains to be determined, and knock-out mice, as well as specific protease inhibitors, might be useful tools for these endeavors. Previous studies demonstrated that TTSPs play key roles in hormone or growth factor activation, epithelial differentiation, and the initiation of proteolytic cascades [62,63]. The mechanism underlying the effects of up-regulated TTSPs in PEDV-infected piglet small intestine tissues requires further investigation. The inhibitors of TMPRSS2 and MSPL may be potential candidates for treatment of PEDV. Moreover, according to a comparison of the promotion effects of these TTSPs on viral replication and titers in vitro, TMPRSS2 and MSPL were particularly strong, suggesting that members of the hepsin/TMPRSS/enteropeptidase subfamily may activate PEDV emergence due to their specific structure. Additional research is underway to determine whether other members of the hepsin/TMPRSS/enteropeptidase subfamily are able to activate PEDV.

The role of TTSPs in the release of PEDV from infected cells has been reported previously [33], although the mechanism by which TTSPs promote the propagation of animal coronaviruses remains unclear. In this study, to explore this mechanism, we first focused on whether TTSPs (TMPRSS2, HAT, DESC1, and MSPL) activated viral transmission via cell–cell fusion assay, and our results demonstrated that the activating effects of MSPL and TMPRSS2 were more robust than those of the other TTSPs. MSPL exhibited the strongest effect followed by TMPRSS2. It has been suggested that the addition of trypsin mediates cell–cell fusion in PEDV-infected cells [64], thus demonstrating that TMPRSS2 and MSPL exhibit trypsin-like characteristics that facilitate cell–cell fusion. However, it should be noted that the cell–cell fusion assay allows the interaction of cell surfaces on which large amounts of receptors and proteases may be expressed, and therefore it might not fully mirror virus–cell fusion.

Although serine proteases are reportedly involved in PEDV entry, it was previously unclear which of them are most effective [41]. Thus, we used the previously published TTSP inhibitor AEBSF-HCl [41] to assess whether TTSPs (TMPRSS2, HAT, DESC1, and MSPL) activate PEDV entry into cells in the absence of trypsin. We found that viral RNA levels were decreased in a dose-dependent manner following AEBSF-HCl treatment. Additionally, we found that the level of viral mRNA increased in a dose-dependent manner in Vero cells expressing TMPRSS2 and MSPL, but not HAT and DESC1 at the stage of virus entry [65]. These results suggest that the activating effect of TMPRSS2 and, in particular, MSPL, on PEDV entry into cells was greater than that of HAT and DESC1. Although Liu et al. found that TMPRSS2 did not increase the entry of PEDV pseudoviruses into Huh-7 cells [66], several studies suggested that some candidate cellular enzymes, such as TTSPs could activate PEDV replication [41,64], and that human TMPRSS2 has been shown to enhance the multicycle replication of PEDV [33].

To explore the key role of TTSPs in facilitating PEDV replication, we speculated that the S protein of PEDV might have interacted with TTSPs located on the cell surface during viral infection. Thus, an assessment of the co-localization of TTSPs with the PEDV S protein was performed, and our results showed that the PEDV S protein co-localized extensively with MSPL and TMPRSS2, indicating that these TTSPs might interact with the PEDV S protein to promote viral entry into cells. It is worth noting that PEDV-activating TTSPs co-localized with S protein, whereas inactive TTSPs did not, despite robust expression (such as DESC1) in the cellular system analyzed. It is therefore conceivable that the cellular localization of a TTSP, apart from its substrate specificity, might determine whether the protease can activate S and other viral glycoproteins; this possibility deserves further investigation. It has been confirmed that TTSPs cleave and activate the SARS-CoV and MERS-CoV S proteins, and the cleaved fragments of the S protein may induce subtle conformational changes that increase its sensitivity for binding to its receptor [15]. We also attempted to verify the cleavage of S protein by TTSPs, and we found TMPRSS2 and MSPL could cleave PEDV S protein with the same size of cleavage fragments, but HAT and DESC1 could not. Therefore, our study further confirmed the roles of TMPRSS2 and MSPL in the PEDV S protein activation. However, the mechanisms of S protein activation by TTSPs for PEDV entry have not been clearly demonstrated, and additional research is under way to further investigate these mechanisms.

Currently, the propagation of PEDV in vitro remains a continuing challenge, as viral infectivity gradually declines during serial passages in cell cultures. In this study, we confirmed that TMPRSS2 and MSPL effectively facilitate the isolation of PEDV in vitro in the absence of trypsin. Viral adaptation and growth in Vero cells expressing TMPRSS2 and MSPL were higher than those in control cells transfected with empty plasmid control and in cells treated with trypsin. These results indicate that TMPRSS2 and MSPL might be more conducive to PEDV isolation in vitro than exogenous proteases like trypsin, suggesting a promising approach for PEDV isolation in vitro, using Vero cell lines continuously expressing TMPRSS2 or MSPL. The establishment of Vero cell lines stably expressing TMPRSS2/MSPL may facilitate the use of attenuated cell-culture-adapted PEDV strains cultured in the absence of trypsin for vaccine development, which can reduce the cost and simplify the process in the PEDV vaccine production.

In conclusion, we first demonstrated that TMPRSS2 and MSPL facilitate the replication of the animal coronavirus PEDV and play a significant role in viral infection by promoting cell–cell fusion and virus–cell fusion. Whether or not the endogenous presence of TTSPs in the small intestines of piglets contributes to viral spread in infected piglets should be determined further. This study provides insights and a novel method for enhancing viral titers, expanding virus production, and improving the adaptability of PEDV isolates in vitro.

## Figures and Tables

**Table 1:** Primers used in the study.

Primers	Primer Sequence (5′→3′)	Targets (ID)
**Primers for the construction of TTSPs plasmids**		
TMPRSS2-F	CCGGAATTCGGATGGCTTTGAACTCAGGG	TMPRSS2
TMPRSS2-R	GGAAGATCTTTAGCCGTCTGCCCTCAT	(BC051839)
HAT-F	CCGGAATTCGGATGTATAGGCCAGCACG	HAT
HAT-R	GGAAGATCTCTAGATCCCAGTTTGTTG	(BC125195)
DESC1-F	CCGGAATTCGGATGATGTATCGGCCAGATG	DESC1
DESC1-R	GGAAGATCTTTAGATACCAGTTTTTG	(BC113412)
MSPL-F	CCGGAATTCGGATGGAGAGGGACAGCC	MSPL
MSPL-R	GGAAGATCTTTAGGATTTTCTGAATCG	(BC114928)
**Primers for identification of PEDV by real-time PCR**		
PN-F	ACTGAGGGTGTTTTCTGGGTTGC	Nucleocapsid gene of PEDV
PN-R	GGTTCAACAATCTCAACTACACTGG	(DQ072726)
Beta-actin-F	AAGGATTCATATGTGGGCGATG	β-actin gene of Vero cells
Beta-actin-R	TCTCCATGTCGTCCCAGTTGGT	(AB004047)
**Primers for identification of swine TTSPs mRNA by real-time PCR**		
sw-TMPRSS2-F	CACCCGAACTATGACCCCAAGACC	Swine-TMPRSS2
sw-TMPRSS2-R	CATAGCGGCGTTCAGCACCTC	(XM_013982601)
sw-HAT-F	ACAACGCACAATAACTCCCTCTG	Swine-HAT
sw-HAT-R	GACATTGTTCTGTTGAAGGCTGG	(XM_013978756)
sw-DESC1-F	TGCTGCTGATTTTTAGATTTCGCTC	Swine-DESC1
sw-DESC1-R	AGGGGGTCCTACAGCATCTTG	(XM_013978755)
sw-MSPL-F	CCCATAAGTGGCTTCCCGTC	Swine-MSPL
sw-MSPL-R	TGTAGATGCTCTCCTGGATGGTG	(XM_013989517)
sw-GAPDH-F	AAGGTCGGAGTGAACGGATTTG	Swine-GAPDH
sw-GAPDH-R	GCCTTGACTGTGCCGTGGAAC	(XM_013991162)
**Primers for identification of TTSPs in Vero cells**		
m-TMPRSS2-F	ACCGCCAGGTGTTGGACCTTAC	m-TMPRSS2
m-TMPRSS2-R	GACACGCCATCGCACCAGTTAG	(XM_007968781)
m-HAT-F	AGTGTGTGTCTCCCAGCTGCTAC	m-HAT
m-HAT-R	TCGGTAGGTTGTCACTCGGGTAT	(XM_007998573)
m-DESC1-F	GGTGGAACAGAAGTAGAAGAGGG	m-DESC1
m-DESC1-R	CACATCACCTGGGTGAAACTC	(XM_007998564)
m-MSPL-F	TGACCCTGTCCGCTCACATCCAC	m-MSPL
m-MSPL-R	AAATCGCACCTCACTCTCCATCTTG	(XM_008021030)
